# TPMS Microarchitectures for Vertical Bone Augmentation and Osteoconduction: An In Vivo Study

**DOI:** 10.3390/ma17112533

**Published:** 2024-05-24

**Authors:** Ekaterina Maevskaia, Chafik Ghayor, Indranil Bhattacharya, Julien Guerrero, Franz E. Weber

**Affiliations:** 1 Center of Dental Medicine, Oral Biotechnology & Bioengineering, University of Zurich, 8032 Zurich, Switzerlandjulien.guerrero@usz.ch (J.G.); 2 Center for Surgical Research, University Hospital and University of Zurich, 8032 Zurich, Switzerland; 3 Center for Applied Biotechnology and Molecular Medicine (CABMM), University of Zurich, 8032 Zurich, Switzerland

**Keywords:** TPMS, microarchitecture, vertical bone augmentation, osteoconduction, bone substitute, additive manufacturing, 3D printing, ceramics

## Abstract

Triply periodic minimal surface microarchitectures (TPMS) were developed by mathematicians and evolved in all kingdoms of living organisms. Renowned for their lightweight yet robust attributes, TPMS structures find application in diverse fields, such as the construction of satellites, aircrafts, and electric vehicles. Moreover, these microarchitectures, despite their intricate geometric patterns, demonstrate potential for application as bone substitutes, despite the inherent gothic style of natural bone microarchitecture. Here, we produced three TPMS microarchitectures, D-diamond, G-gyroid, and P-primitive, by 3D printing from hydroxyapatite. We explored their mechanical characterization and, further, implanted them to study their bone augmentation and osteoconduction potential. In terms of strength, the D-diamond and G-gyroid performed significantly better than the P-primitive. In a calvarial defect model and a calvarial bone augmentation model, where osteoconduction is determined as the extent of bony bridging of the defect and bone augmentation as the maximal vertical bone ingrowth, the G-gyroid performed significantly better than the P-primitive. No significant difference in performance was observed between the G-gyroid and D-diamond. Since, in real life, the treatment of bone deficiencies in patients comprises elements of defect bridging and bone augmentation, ceramic scaffolds with D-diamond and G-gyroid microarchitectures appear as the best choice for a TPMS-based scaffold in bone tissue engineering.

## 1. Introduction

Vertical bone augmentation, a frequently applied procedure in dentistry and oral implantology, aims at increasing the height of the alveolar ridge to accommodate dental implants. It is commonly employed when the available bone height is insufficient due to trauma, periodontal disease, or tooth loss. Over the last few decades, numerous studies have been conducted to explore various approaches and materials for vertical bone augmentation [1]. Although bone augmentation procedures have proved efficient for the reconstruction of the alveolar bone, the optimal procedure and material remain the subject of ongoing debates [1].

Osteoconduction is a fundamental concept in bone tissue engineering and regenerative medicine. It refers to the ability of a scaffold to provide a supportive framework or structure for the ingrowth of new bone tissue [2]. In contrast to bone augmentation, where bone ingrowth occurs from one side only, in osteoconduction, the scaffold is placed in a bone defect, providing a bony bed from which bone ingrowth occurs from all sides in contact with bone tissue.

Both procedures rely heavily on the use of a scaffold to serve as a placeholder and as a guiding cue for the newly-forming bone. The gold-standard scaffold for both procedures is still autologous bone [2,3], although, it has obvious limitations related to its volume availability and to increased patient morbidity [4]. In spite of there being a high variety of materials proposed to replace the application of autografts, there is currently no clear evidence on which bone substitute is more effective for different bone regenerative purposes in the oral cavity [5]. The structure of the trabecular-organized cancellous bone resembles a gothic style of architecture [6] and provides the bone with high strength at a low weight since the local load is taken up by individual trabecula. However, when transferred as a block, the microarchitecture of the autologous transplant is optimized for the donor’s site load according to Wolff’s law [7], necessitating subsequent adjustment through bone remodeling at the recipient site [7]. To overcome this limitation, triply periodic minimal surface (TPMS)-based architectures can be applied more broadly since they are designed to fill volumes by providing the overall structure with high strength at low weight. Therefore, TPMS designs can be found in aircrafts, satellites, and electric vehicles to minimize material usage and fuel consumption [8].

In industry, lightweight components are often designed with algorithms to generate honeycomb-based porous microarchitectures [9] or TPMS microarchitectures [10]. The honeycomb design was inspired by nature. The TPMS microarchitectures P-primitive and D-diamond were developed while solving a mathematical problem and were only later found to be present in nature. The mathematical problem was posed in 1865 by the “königliche Akademie der Wissenschaften zu Berlin” (Germany) to find the minimal surface formed between four points, each located on one of the four sides of a tetrahedron [11]. The third TPMS algorithm leading to the G-gyroid structure was developed in 1970 [12]. TPMS architectures are lightweight due to their minimal surface requirement and high strength, which is because the average curvature at each point on the surface is zero and the surfaces are devoid of self-intersections. Therefore, they appear suitable for bone substitutes. In addition, TPMS architectures partition the space into two independent labyrinths [13]. The identification of a G-surface in nature at the interface between the inorganic crystalline and organic amorphous matter in echinoderm plates in 1965 [14] and subsequent recognition of G-, D-, and P-structures in butterfly wings, in the exoskeleton of beetles, and ultimately in all the kingdoms of living organisms [13] took several decades.

Salt leaching, gas foaming, and phase separation followed by freeze-drying are used to produce porous materials [15]. The resulting microarchitectures, however, are unpredictable [2]. In the context of bone tissue engineering, the microarchitecture of a bone substitute defines pores and interconnections as it represents the distribution of the material in the macroarchitecture, which occupies the space of the bone defect [2]. The advent of additive manufacturing has revolutionized the production of highly defined porous, personalized bone substitutes for bone tissue engineering [2], including the highly complex TPMS microarchitectures [16] and the adaptive density minimal surface (ADMS) microarchitectures [17]. Additive manufacturing also facilitated the production of honeycomb-based scaffolds for bone augmentation [18]. Notably, 3D-printed filament-based microarchitectures have demonstrated superior bone augmentation outcomes compared to particle-based random designs [19,20].

While G-gyroid, D-diamond, and P-primitive have been mainly tested in silico or in vitro for bone tissue engineering, limited in vivo data exist for TPMS microarchitectures. For G-gyroid microarchitecture, unit cell number and wall thickness turned out to be crucial factors in controlling the modulus, maximum stress, and energy absorption [21]. Its higher permeability compared to P-primitive and D-diamond scaffolds could prove advantageous in cell seeding efficiency, cellular infiltration, differentiation, and new tissue formation in vivo [22]. The D-diamond design exhibited superior performance in terms of strength, elasticity, and energy absorption [23].

In vivo results with TPMS microarchitectures are scarce. Good osseointegration, regardless of pore size, was seen with P-primitive microarchitectures [24]. Compared to cross-hatch, lattice-like structures, the P-primitive showed higher osteoconductivity and bone metabolic activity [25] in a rat calvaria defect model [26]. D-diamond designs of titanium scaffolds yielded good bone regeneration in femurs but poor results in cranial defects [27]. Nevertheless, to our knowledge, there are no comparative studies with scaffolds based on different types of TPMS microarchitectures besides our recent study on osteoconduction of TPMS microarchitectures with a common bottleneck diameter of 0.8 mm [16].

The aim of this study was to determine whether the optimal TPMS microarchitecture for osteoconduction aligns with that for bone augmentation. For that, an in-depth analysis of the in vivo performance of D-diamond, G-gyroid, and P-primitive with a common bottleneck diameter of 0.8 mm in a rabbit model for bone augmentation and a one-sided ingrowth model in cranial defects was performed. Additionally, the scaffolds were characterized by their mechanical properties.

## 2. Materials and Methods

### 2.1. Materials

#### Manufacturing of Scaffolds

The HA-based scaffolds were produced with the aid of a lithography-based 3D printer for ceramics (CeraFab 7500, Lithoz, Vienna, Austria), using the photosensitive hydroxyapatite-based slurry LithaBone™ HA 400 (Lithoz, Austria) to produce green bodies. The corresponding stl-files, coding for the scaffolds, were designed with the nTopology v.3 software (USA). The wall thickness was 0.2 mm for all TPMS designs and the minimal constriction was fixed at 0.8 mm. After cleaning the 3D-printed scaffolds with pressured air and LithaSol 30™ (Lithoz, Vienna, Austria), they were debindered and sintered using the cycle presented in Table 1.

### 2.2. Methods

#### 2.2.1. Mechanical Testing

Mechanical properties were measured by applying compressive load at a speed of 1 mm/min in the direction of building layers (universal testing machine ROELL Z2.5 MA 18-1-3/7, Zwick, Ulm, Germany). For this purpose, cubic scaffolds 7.8 × 7.8 × 7.8 mm^3^ of the three TPMS microarchitectures were designed and produced. A minimum of 8 scaffolds of each microarchitecture were used for the mechanical testing. The maximal compression force was determined from the stress–strain curve at the breaking point using TestXpert V11.02 software (Zwick, Germany).

#### 2.2.2. Surgical Procedure for 1-Sided Bone Ingrowth

**Figure 1:**
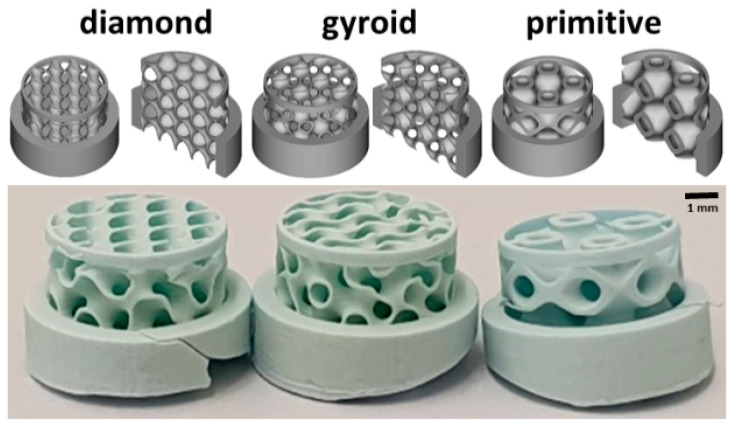
Micro- and macroarchitecture of the scaffolds used in the osteoconduction/bone defect model. In the upper panel, the overall macroarchitectures of halved and full scaffolds are illustrated. In the lower panel, the constructs are displayed. A scale bar is provided.

Scaffolds with the three TPMS microarchitectures used for the determination of 1-sided bone ingrowth had a diameter of 6.0 mm and a height of 5.0 mm. To restrict their advancement beyond the thickness of the calvarial bone, a solid outer ring with a 6.0 mm inner, 7.5 mm outer diameter, and a height of 2.5 mm was added to the upper part. Therefore, the TPMS microarchitectures could emerge into the calvarial bone by 2.5 mm only. To stabilize the lower part, another ring with an outer diameter of 6.0 mm, an inner diameter of 5.7 mm, and 0.5 mm in height was added to the TPMS microarchitecture (Figure 1). A total of 8 scaffolds of each microarchitecture were implanted.

All animal procedures were approved by the Animal Ethics Committee of the local authorities (Canton Zurich, 065/2018 and 090/2021) and performed following the ethics criteria contained in the bylaws of the Institutional Animal Care and Use Committee. The procedure was carried out as described earlier [28]. Briefly, the scaffolds were inserted into calvarial defects of 12 rabbits (female, 26-week-old, New Zealand white rabbit). Three TPMS-based scaffolds and one experimental scaffold were implanted per animal. Four weeks after the implantation, the animals were first anesthetized and then sacrificed by an overdose of pentobarbital.

#### 2.2.3. Surgical Procedure for Bone Augmentation

**Figure 2:**
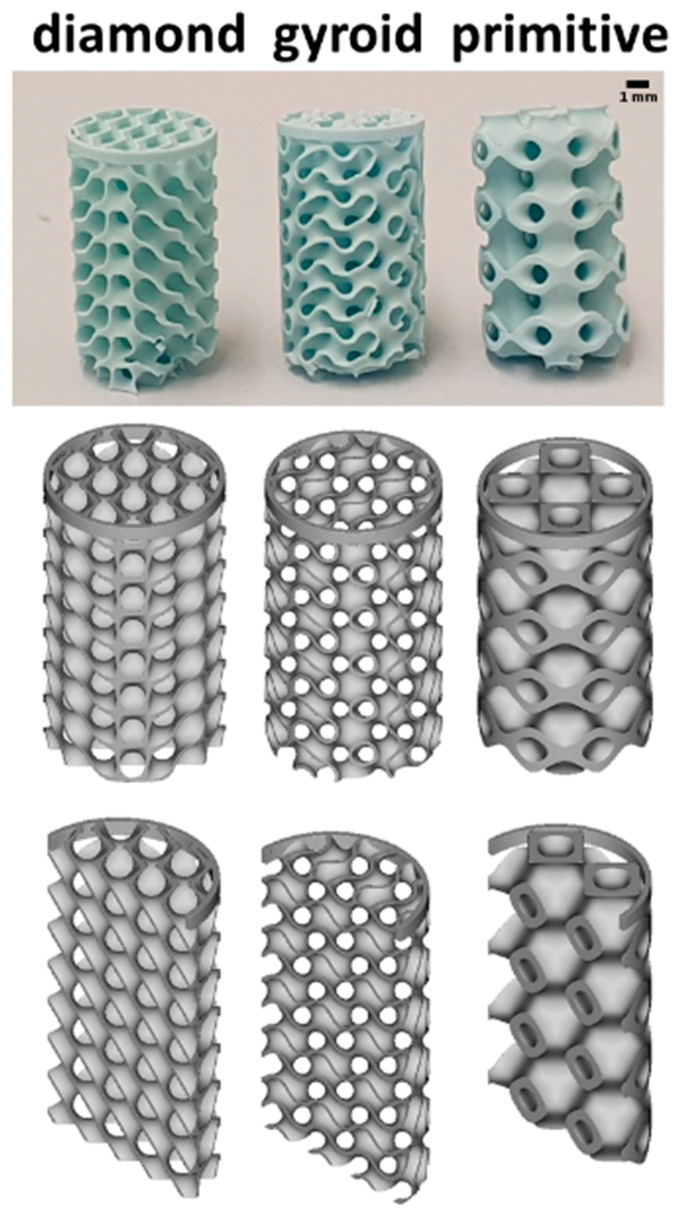
Micro- and macroarchitecture of the scaffolds used in the bone augmentation model. In the upper panel, the constructs are displayed. A scale bar is provided. In the lower two panels, the overall macroarchitecture of full and halved scaffolds is illustrated.

Scaffolds with the three TPMS microarchitectures used for the assessment of bone augmentation had a diameter of 6.00 mm and a height of 12.00 mm. The lower part of the implants was stabilized by a ring with an outer diameter of 6.00 mm, an inner diameter of 5.70 mm, and measuring 0.50 mm in height (Figure 2). A total of 8 scaffolds of each microarchitecture were implanted.

**Figure 3:**
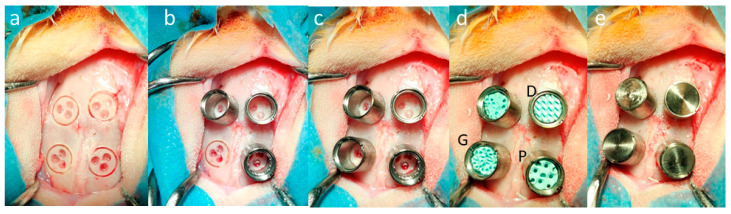
Calvarial model for bone augmentation: (**a**) the four sites for cylinder placement were prepared with a trephine, and bone ingrowth was primed by a bur to generate three small defects in the outer cortical plate of the calvarial bone; (**b**,**c**) the four cylinders were screwed into the circular slits; (**d**) scaffolds were inserted (D, P, and G in black indicates the position of D-diamond, G-gyroid, and P-primitive); and (**e**) the cylinders were closed by press-fitting titanium lids. The diameter of the circular slits (**a**,**b**) is 6.00 mm.

This procedure was also approved by the Animal Ethics Committee of the local authorities (Canton Zurich, 065/2018 and 090/2021) and performed following the ethics criteria contained in the bylaws of the Institutional Animal Care and Use Committee. Bone augmentation in in vivo model was described earlier [29]. In brief: four evenly distributed circular slits of 6.00 mm diameter and 1.00 mm sink depth were created with a trephine. Next, the external cortical plate inside the circles was perforated three times with a 1.00 mm burr. After rinsing the operation site, four titanium cylinders (7.00 mm in height and 7.00 mm in outer diameter) were screwed into the prepared slits, providing primary stability. The cylinders were filled with the three TPMS-based scaffolds plus an experimental scaffold. Just before the transfer into the cylinders, the scaffolds were shortened with a scalpel to 5.00 mm, closed with a titanium lid, and the skin sutured to cover the calvarial bone and the cylinders (Figure 3).

Four weeks after the operation, the rabbits were administered general anesthesia and were sacrificed using an overdose of pentobarbital. The cranium containing all four cylinders was removed and embedded.

#### 2.2.4. Histomorphometry

The excised calvaria containing the scaffolds were embedded in methyl methacrylate (MMA) and divided into four pieces, each containing either an implant or a cylinder. From all samples, ground sections from the middle of the implant or the cylinder were prepared, stained with toluidine blue to visualize bone tissue, and photographed using image analysis software (Image-Pro Plus^®^; V11, Media Cybernetic, Silver Springs, MD, USA). The maximal stretch of bone ingrowth was determined from the lower end of the cylinder towards the lid for bone augmentation and from each side of the bone defect margins for osteoconduction, respectively. The area of bony regeneration is the percentage of bone and bone-integrated scaffold in the cylinder for bone augmentation and the half of the area between the defect margins and the 2.50 mm restricted sink depth of the scaffolds for osteoconduction, respectively.

#### 2.2.5. Statistics

For osteoconduction into the calvarial defect, the ingrowth of the bone front was determined from the left and the right defect margin in the ground section. Therefore, each half of the defect and the cylinder represented the primary analysis unit. Data from 6 to 11 different rabbits were compiled in each group and were composed of 8–12 samples. IBM SPSS Statistics version 19.0 (IBM Corp., Armonk, NY, USA) was used to compare groups first by the Kruskal–Wallis test for both measures, followed by pairwise comparison of treatment modalities using the Mann–Whitney test for independent data. *p*-values below 0.05 were defined as statistically significant. *p*-values are displayed in the graphs and the data are presented in the text as mean ± standard deviation or as median ± lower/upper quartile.

## 3. Results

### 3.1. Compression Strength of HA-Based TPMS Microarchitectures

**Figure 4:**
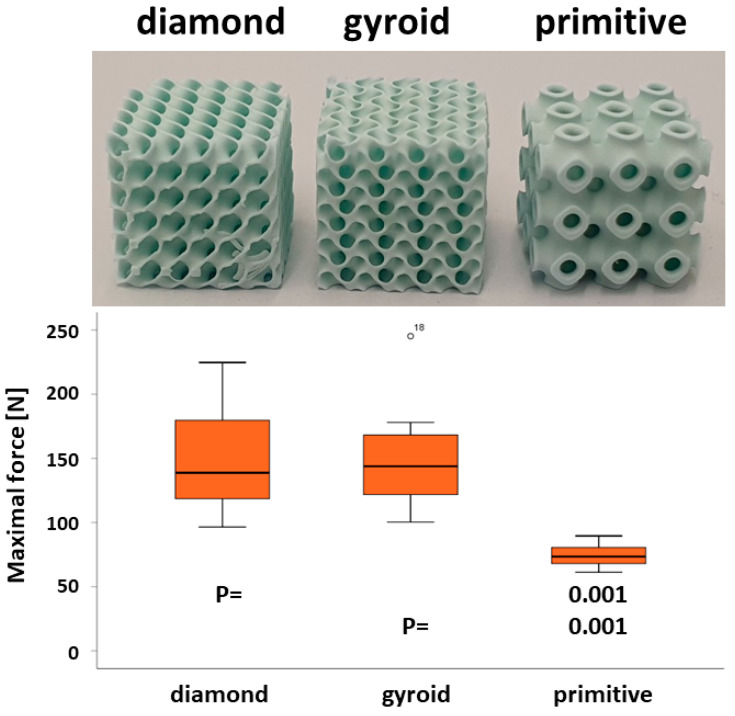
Mechanical testing of TPMS microarchitectures. Cubic scaffolds 7.80 × 7.80 × 7.80 mm^3^ of the three TPMS microarchitectures were tested (upper panel). The graph in the lower panel visualizes the results of the testing for the maximal force in a box plot ranging from the 25th (lower quartile) to the 75th (upper quartile) percentile, with the median displayed as a solid black line and whiskers extending to the minimum and maximum values. Individual points outside the range are also displayed. The displayed *p*-values (P) show the significance level between the D-diamond and P-primitive scaffolds (upper) and between the G-gyroid and P-primitive scaffolds (lower). The compression strength of D-diamond and G-gyroid are significantly higher than for P-primitive.

The mechanical properties were determined under a compressive load. The maximal force before failure which could be applied to the D-diamond cube was 146.67 ± 41.81 N, for the G-gyroid 151.66 ± 45.11 N, and 74.37 ± 8.91 N for the P-primitive, respectively (Figure 4). The D-diamond and G-gyroid resisted significantly higher forces than the P-primitive.

### 3.2. Implantation of HA-Based Scaffolds with TPMS Microarchitecture

All animals used for bone augmentation or osteoconduction remained in good health. Bone formation and lack of signs of inflammation in the defect or the cylinders indicated a good overall biocompatibility of the material and the designs.

#### 3.2.1. Performance of TPMS Microarchitectures in Bone Augmentation

**Figure 5:**
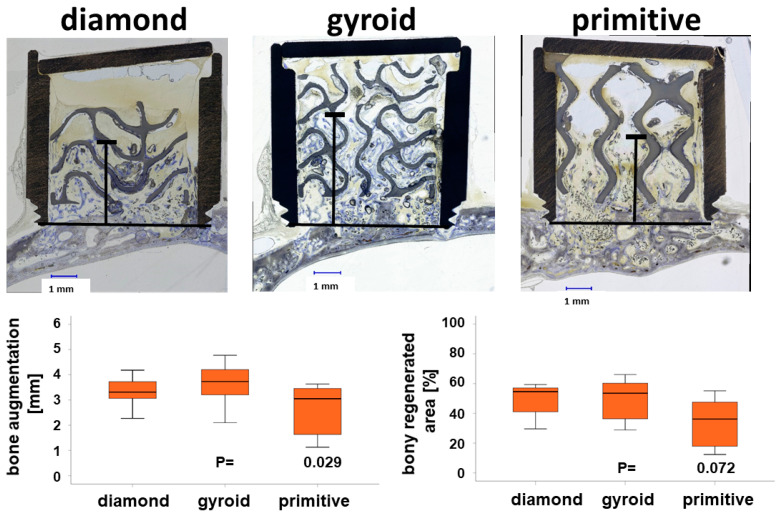
Vertical bone augmentation according to the TPMS microarchitecture of the scaffold. Central histologic sections of cylinders harvested from the crania of rabbits after 4 weeks (upper panel). The highest point of bone ingrowth is depicted by the black line. Titanium appears black, the scaffolds appear greyish, and bone appears greyish purple. Scale bars of 1.00 mm and type of TPMS microarchitecture are provided. Bone augmentation with D-diamond and G-gyroid was significantly higher than for the P-primitive-based microarchitecture (lower panel, left). The bony regenerated area was also higher for D-diamond and G-gyroid compared to P-primitive, however, not to a level of significance. Values are displayed as box plots ranging from the 25th (lower quartile) to the 75th (upper quartile) percentile, with the median displayed as the solid black line and the whiskers extending to the minimum and maximum values. The displayed *p*-values (P) show the significance level between the G-gyroid and P-primitive scaffolds.

An advancement of bone tissue beyond the level of the original calvarial bone occurred for all three TPMS-based microarchitectures. For the D-diamond, bone advancement of 3.33 ± 0.65 mm into the cylinders was observed. The advancement was 3.64 ± 0.84 mm for the G-gyroid and 2.57 ± 1.06 mm for the P-primitive (Figure 5). The G-gyroid scaffolds performed significantly better than the P-primitive in terms of bone augmentation.

The percentage area of bony regeneration was 48.52 ± 11.45% for the D-diamond, 49.40 ± 14.07% for the G-gyroid, and 33.48 ± 17.44% for the P-primitive (Figure 5, lower panel right side). In spite of the lower values obtained for the P-primitive, no significant differences between the scaffolds were found for the bony regenerated area.

#### 3.2.2. Performance of TPMS Microarchitectures in Osteoconduction

Scaffolds with the same TPMS microarchitecture, as used to determine vertical bone augmentation, were used to determine one-sided bone ingrowth as a measure of osteoconductivity.

**Figure 6:**
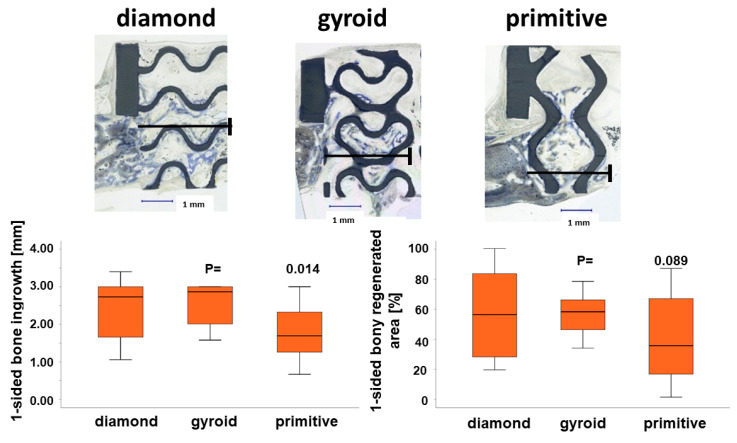
One-sided bone ingrowth according to the TPMS microarchitecture. Central histologic sections of one half of the 6.00 mm defects harvested from the crania of rabbits after 4 weeks are displayed for all three TPMS microarchitectures (upper panel). A scale bar for 1.00 mm is provided. Maximal bone ingrowth is depicted by the black line. The scaffolds appear dark greyish, and bone blue. One-sided bone ingrowth into the 6.00 mm defect was significantly higher in D-diamond and G-gyroid compared to P-primitive (lower panel, left side). Similar results were observed in terms of the percentage area of bony regeneration, although they did not reach a level of significance (lower panel, right side). Values are displayed as box plots ranging from the 25th (lower quartile) to the 75th (upper quartile) percentile, with the median displayed as the solid black line and the whiskers extending to the minimum and maximum values. The displayed *p*-values (P) report the significance level between the G-gyroid and P-primitive scaffolds.

Maximal bone ingrowth from one side into the defect guided by the D-diamond microarchitecture was 2.38 ± 0.76 mm, 2.53 ± 0.54 mm for the G-gyroid, and 1.81 ± 0.72 mm for the scaffolds with the P-primitive microarchitecture. The best TPMS microarchitectures for bone ingrowth into the defect and osteoconductivity were the D-diamond and G-gyroid, and they were significantly superior to the P-primitive (Figure 6, lower panel). No significant difference was reached for the bony regenerated area, although the D-diamond with 56.53 ± 28.01% and the G-gyroid with 57.96 ± 13.53% were substantially higher than the 41.23 ± 30.68% reached with the P-primitive microarchitecture.

For both bone augmentation and osteoconduction, a significant increase in bone ingrowth for the G-gyroid in comparison to the P-primitive was revealed, but no significant differences for bony regenerated area were found. This suggests that although bone formation was the same for all three microarchitectures, the G-gyroid directed bone ingrowth towards the defect bridging and bone augmentation was significantly more efficiently than the P-primitive.

## 4. Discussion

**Figure 7:**
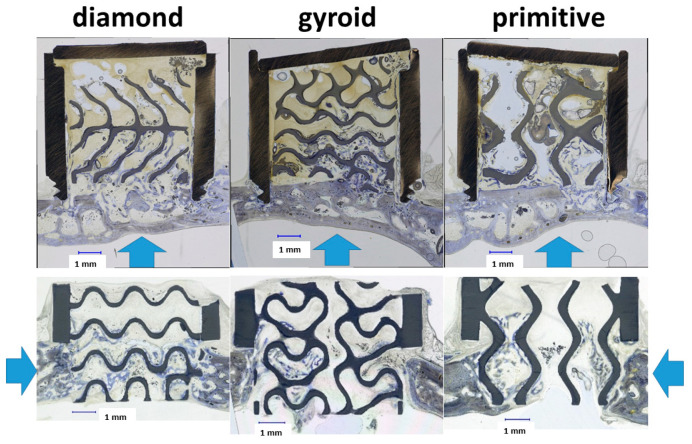
The direction of bone growth in bone augmentation (upper panel) and osteoconduction (lower panel). Ground middle sections derived after 4 weeks of implantation are shown for all three TPMS microarchitectures tested. The direction of bone growth is indicated by blue arrows. Type of TPMS microarchitecture and scale bar of 1.00 mm is provided.

In the present study, we conducted in vivo testing of three TPMS microarchitectures, namely, D-diamond, G-gyroid, and P-primitive, for their suitability to serve as a microarchitecture of a scaffold for bone augmentation and osteoconduction (Figure 7). All three TPMS microarchitectures had in common a bottleneck of 0.80 mm, which we found optimal in lattice architectures [30].

Our findings revealed that in both in vivo applications and the mechanical testing, the G-gyroid performed significantly better than the P-primitive and was on a par with the D-diamond, for which the obtained values were in the range of the compression strength for a cancellous bone (2–8 MPa) [31]. Therefore, the G-gyroid and D-diamond emerge as versatile microarchitectures, ideally suited for various bone repair and bone augmentation applications.

Recently, honeycomb microarchitectures were suggested for bone augmentation applications [18]. Their channels had a diameter of 0.2 mm, and their mechanical performance was tuned by the strut thickness. In terms of bone and vessel ingrowth in vivo, struts with 0.3 mm diameter forming channels of 0.2 mm showed superior performance [18]. The maximal bone advancement in the honeycomb microarchitectures after 4 weeks was 3.1 ± 0.8 mm and in the same range (3.64 ± 0.84 mm), as with our wide-open porous G-gyroid over the same period. Thus, in terms of increase in bone height, honeycomb and the two TPMS microarchitectures, namely the G-gyroid and D-diamond, perform equally well. The major disadvantage of a honeycomb microarchitecture is, however, the uniaxial orientation of the non-interconnected channels, which prevents any bone ingrowth perpendicular to the struts. In that respect, the orientation-independent applicability of the G-gyroid and D-diamond for bone augmentation and osteoconduction (Figure 7) appears, in addition to the lightweight feature, as the major advantage of the TPMS microarchitectures. Moreover, deficiencies in bone height demand rarely for bone augmentation only. In most circumstances, lateral bone ingrowth should occur as well. Therefore, the versatile, bone-ingrowth direction-independent applicability of TPMS microarchitecture is an advantage.

Comparisons with other studies further substantiate the superiority of TPMS microarchitectures. In a sheep calvarial bone augmentation model, a 3D-printed ceramic grid microarchitecture was superior to all other bone substitutes used [19]. In a femoral condyle defect model in rabbits, the G-gyroid was shown to be superior to a filament-based grid structure [32]. However, the pore diameter in both designs was below 0.3 mm and, therefore, too small for fast bone healing by osteoconduction [28]. Moreover, bone ingrowth occurs on the surface of the scaffold if channels are 0.3 mm and below, compared to bone ingrowth occurring between the filaments if channels are 0.5 mm and wider [33]. For a wide-open porous G-gyroid design, as studied herein in the calvarium of rabbits, one-sided bony bridging was 84% (Figure 6), which is not superior to the 78 to 92% extent of bony bridging achieved with diverse filament-based grid architectures in the same test system [34]. This is in line with previous findings that showed that an optimized curvy periodic minimal surface microarchitecture performs as well as a strait optimized lattice microarchitecture [17]. This suggests that in terms of bone ingrowth and healing, a curvy microarchitecture, preferentially the G-gyroid or D-diamond, performs equally well as a strait lattice or grid microarchitecture. The mechanics and the lightweight features of TPMS microarchitectures are, however, superior to grid and lattice microarchitectures [16,35]. Taking into account that graft loss is one of the most frequent complications for vertical augmentation with bone blocks [36], the use of microarchitectures with the enhanced mechanical properties is beneficial.

The porosity of the scaffold influences the tissue ingrowth. To be in the range of natural bone, it should be greater than 50% [37], and is met by all our scaffolds. Scaffolds within a smaller range of 72.3% < porosity < 88.4% were shown to be highly osteoconductive [38]. As only porosity values for the P-primitive scaffold are not in that range, it could be one of the possible reasons for the superior results obtained with the G-gyroid and D-diamond microarchitectures. For bone augmentation, uniform pores of 1.70 mm perform best, while for bone defects a diameter of 1.20 mm is preferable [29]. The maximal diameter of a 2D sphere fitting in the microarchitecture displayed on ground sections for the P-primitive is 1.65 ± 0.12 mm (Table 2). The values for the G-gyroid and D-diamond are below 1.00 mm. Since the G-gyroid performs significantly better in bone augmentation than the P-primitive, this suggests that single pores of 1.65 mm are not sufficient to grant a superior bone augmentation performance compared to pores measuring between 0.80 and 1.00 mm in diameter. Overall, G-gyroid and D-diamond TPMS microarchitectures are best suited for both applications and let them appear as ideal, all-purpose bone substitute microarchitectures in bone tissue engineering. Moreover, G-gyroid hydroxyapatite scaffolds were associated with slightly higher levels of osteointegration and appositional mineralization in a sheep model than the commercially available bone substitute Bio-Oss^®^ [39]. In that case, a femoral condyle model was used in contrast to the cranial model used in our study. To further investigate the potential of TPMS-based bone grafts, other animal models or critical size defects should be performed.

For future research, the influence of TPMS microarchitecture parameters on their suitability for osteoconduction and augmentation procedures should be studied. It was shown that tuning the pore size and interconnectivity of TPMS scaffolds can boost the differentiation of pre-osteoblastic cell lines [40]. Regarding the optimal pore size, results are inconclusive. Earlier, the pore size of 325 μm was considered to be optimal for bone tissue engineering [41]. Later, the range was increased, and it was shown that macroporous (100 and 600 μm) scaffolds allow better integration with the host bone tissue, subsequent vascularization, and bone distribution [42]. An average pore size of 550 µm was shown to be optimal for bone formation [43]. To clarify the existing controversies, a library of 15 scaffolds with diverse defined pore/bottleneck dimensions and distributions was produced. It was shown that the ideal pore/bottleneck dimension for bone substitutes is in the range of 0.7–1.2 mm [28]. However, the microarchitecture of the scaffolds also varies between different studies, therefore, it would be beneficial to explore the influence of pore size on the osteoconductive properties of scaffolds specifically for TPMS architectures further.

## 5. Conclusions

Among the evaluated TPMS microarchitectures, ceramic scaffolds featuring G-gyroid and D-diamond microarchitectures exhibit superior suitability for bone augmentation and defect treatment and have higher strength compared to the P-primitive. Therefore, beyond their inherent lightweight, high-strength characteristics, scaffolds with G-gyroid and D-diamond microarchitectures emerge as superior and universal treatment options, addressing various challenges in bone tissue engineering.

## Figures and Tables

**Table 1:** Sintering cycle.

Heating Time, hh:mm	Heating Rate, K/min	Temperature, °C	Holding Time, h
-	-	25	2
02:00	0.42	75	2
04:00	0.17	115	4
08:00	0.19	205	16
20:00	0.19	430	4
06:00	0.47	600	0
08:00	0.52	850	2
07:42	0.97	1300	2
13:01	−1.62	30	0

**Table 2:** Architectural features of TPMS microarchitectures.

Characteristics of TPMS Microarchitectures	D-Diamond	G-Gyroid	P-Primitive
Bottleneck [mm]	0.80	0.80	0.80
Microporosity [%]	82.00	79.00	69.00
Maximal diameter of 2D sphere fitting in microarchitecture [mm]	0.99 ± 0.05	0.76 ± 0.05	1.65 ± 0.12

## Data Availability

The raw/processed data required to reproduce these findings cannot be shared at this time as the data also form part of additional ongoing studies.
